# Design of an MIP-Based Electrochemical Sensor for the Determination of Paracetamol in Pharmaceutical Samples

**DOI:** 10.3390/bios15080544

**Published:** 2025-08-19

**Authors:** José Alberto Cabas Rodríguez, Fernando Javier Arévalo, Adrian Marcelo Granero

**Affiliations:** Departamento de Química, Grupo GEANA, Instituto para el Desarrollo Agroindustrial y de la Salud (IDAS), Facultad de Ciencias Exactas, Fisicoquímicas y Naturales, Universidad Nacional de Río Cuarto, Agencia Postal No 3, Río Cuarto 5800, Argentina; jcabasrodriguez@exa.unrc.edu.ar

**Keywords:** electrochemical sensor, pharmaceutical samples, paracetamol, molecularly imprinted polymer

## Abstract

Paracetamol (PAR) is a common antipyretic and analgesic extensively used to treat cold and flu symptoms. It has been proven to be effective in headaches and relieving fever and pain. It is usually found as an over-the-counter drug, which has been associated with an increase in cases of poisoning due to overdose. Therefore, the development of new analytical tools for the detection of PAR at low concentrations in different samples is necessary. In this work, a Molecularly Imprinted Polymer (MIP)-based electrochemical sensor was designed for the selective and sensitive determination of PAR using a glassy carbon electrode (GCE) modified with a polymeric film obtained through the electropolymerization of o-aminophenol. A complete characterization based on electrochemical techniques, such as electrochemical impedance spectroscopy (EIS) and cyclic voltammetry (CV), and scanning electron microscopy (SEM) was used to examine all steps involved in the construction of the MIP-based electrochemical sensor. In addition, all parameters affecting the MIP were optimized. As a result, the MIP-based electrochemical sensor showed a very low limit of detection (LOD) of 10 nM, with an analytical sensitivity of (3.4 ± 0.1) A M⁻¹. In addition, construction of the MIP-based electrochemical sensor showed highly reproducibility, expressed in terms of a variation coefficient lower than 4%. The MIP-based electrochemical sensor was successfully used in an assay for the determination of PAR in pharmaceutical products. The performance of the MIP-based electrochemical sensor was compared to High Performance Liquid Chromatography (HPLC) for the determination of PAR in pharmaceutical samples, showing excellent agreement between the two methodologies. A very important aspect of the developed sensor was its reusability for at least twenty times. The MIP-based electrochemical sensor is a reliable analytical tool for the determination of PAR.

## 1. Introduction

Paracetamol (PAR), also known as acetaminophen, is a widely used non-steroidal anti-inflammatory drug widely used for its efficacy as an analgesic in the treatment of the pain and as a fever reducer, as recommended by the World Health Organization [1]. Generally, paracetamol has no health adverse effects. However, despite its recognized therapeutic benefits, high-dose usage has been associated with adverse effects, primarily renal and hepatic damage, due to the accumulation of toxic metabolites [2]. On the other hand, paracetamol is classified as an over-the-counter drug in most countries, contributing to its widespread accessibility and use. Therefore, there is excessive and abusive consumption. Consequently, it is imperative that precise and reliable analytical methods are developed and implemented for monitoring its presence in biological and environmental samples [3]. In this context, there are widely used techniques to quantify paracetamol, such as HPLC [4], gas chromatography [5], thin layer chromatography [6], chemiluminescence [7], UV-visible spectroscopy [8,9], and mass spectroscopy [10]. However, most of these methods require expensive equipment, long processing times, and high reagent consumption [11]. As a consequence, the development of new methods that will be faster, less expensive, and reliable is of interest. In this sense, electroanalytical sensors are a promising option due to their simplicity, speed of analysis, and the capacity to miniaturize the system [12]. Electroanalytical techniques play a significant role in food security and quality control in the pharmaceutical industry [13,14]. In this sense, it is known that electroanalytical techniques based on potential pulses are very sensitive. Therefore, electrochemical sensors are a reliable option for determining a wide variety of molecules. However, selectivity challenges may arise when analyzing complex matrices, wherein existing molecules can prove to be sources of interference [15].

PAR is a well-characterized electroactive compound whose anodic oxidation mechanism proceeds via a two-electron (2 e^−^) and two-proton (2 H^+^) transfer pathway, with its voltammetric behavior exhibiting a pronounced dependence on the solution pH [16]. A wide range of electrochemical platforms have been engineered for its quantitative determination in pharmaceutical formulations, such as poly(Co(II)-phenanthroline, diresorcinate)-modified electrodes [17], AgNPs@HOOC-MWCNT nanocomposites [18], and polyimide-multi-walled carbon nanotubes membrane-based sensors [19]. These architectures synergistically combine high surface conductivity with structural stability, leading to improved analytical sensitivity and operational robustness in complex matrices.

Despite these advances, achieving high selectivity remains a persistent challenge, primarily due to the co-existence in pharmaceutical products of other redox-active compounds, including ascorbic acid (AA), diclofenac (DS), and ibuprofen (Ib), which may produce overlapping oxidation peaks or undergo competitive adsorption at the sensing interface, thereby undermining the accuracy of quantitative analysis [20,21,22].

To address these limitations, molecularly imprinted polymers (MIPs) have emerged as a powerful strategy, offering structurally defined recognition sites that are complementary to the target analyte [23,24,25]. MIPs show higher affinity and selectivity for a specific target, comparable to biomolecules such as enzymes and antibodies. In fact, MIPs mimic the molecular recognition of biomolecules [26]. Notable examples include an MIP-(PCz-co-PPy)/AuPd/GN-CNTs-IL sensor, where a glassy carbon electrode was modified with a nanocomposite of AuPd nanoparticles and ionic liquid-functionalized graphene-carbon nanotubes [27]. Another instance is NanoMIPs/SPCE, which employs screen-printed carbon electrodes coated with electroactive molecularly imprinted polymer nanoparticles synthesized using itaconic acid as the functional monomer [28]. Also reported is the ZrO₂-MIP-Au/rGO device, which integrates Au nanoparticle-modified zirconia inorganic molecularly imprinted membranes on reduced graphene oxide [29]. The MIP/MoS₂/CNTs/GCE platform incorporates a hybrid 1T/2H MoS₂/multi-walled carbon nanotube composite onto a glassy carbon electrode [30], while the MMIP/MCPE configuration utilizes a magnetic surface molecularly imprinted membrane within a modified carbon paste electrode [31]. Finally, the MIP/GO@COF/GCE sensor combines a graphene oxide@covalent organic framework nanocomposite with a glassy carbon electrode [32]. Collectively, these systems exhibit remarkable molecular selectivity and strong anti-interference performance in varied analytical contexts.

MIP production is based on the polymerization reaction on a specific monomer or mix of monomers in presence of a molecule target, where the polymer matrix formed contains the target molecule within its structure. Various techniques for synthesizing MIPs have been explored, including chemical polymerization, sol–gel methods, photopolymerization, and electropolymerization [33]. Electrosynthesis is a highly efficient method for making these polymers, enabling precise control over the structure and properties of the printed polymer through adjustment of the electrochemical parameters during their synthesis [34]. The MIP recognizes the target molecule through specific interactions-including hydrogen bonding, van der Waals forces, and electrostatic interactions-established during the polymerization process. These labile interactions allow, in a subsequent step (extraction step), to remove the target of the polymer forming the MIP, which has specific cavities. Cavities have a unique spatial configuration with specific functional groups, which allows to the MIP to recognize the molecule target with a high specificity level [35]. Therefore, MIPs are used to develop selective electrochemical sensors [36,37].

The monomer used to develop the MIP should contain functional groups (e.g., nitrogen-, oxygen-, or sulfur-based groups) that can interact with the target molecule. Thus, among the most commonly used monomers for the synthesis of molecularly imprinted polymers (MIPs) via electropolymerization are pyrrole [38], o-aminophenol [39], and a wide variety of other monomers, which are described in detail in the bibliography [40,41], due to their ability to interact specifically with the template molecule and form its derivate conductive polymers. These polymers exhibit good compatibility with electrode surfaces, facilitating electron transfer in electrochemical sensors. On the other hand, monomers such as methacrylic acid [42] and itaconic acid [43], which lack conjugated systems, yield non-conductive polymers. Although these materials do not enhance the conductivity of the system, they are widely used due to their high affinity and ability to form specific interactions with the target analyte. Therefore, they are often employed in combination with functional monomers or conductive matrices to form copolymers, aiming to balance molecular selectivity with the electrochemical performance of the sensor.

However, when an electrochemical electrode is modified with a polymer, the electrocatalytic behavior of the electrode decreases depending on the conductivity of the polymer. It is imperative to overcome this problem to obtain a sensitive electrochemical sensor. In this sense, electrodes modified with nanomaterials based on carbon [44], quantum dots [45], and metallic nanoparticles [46] are used to increase the conductivity of electrodes modified with polymers. Graphene and its derivatives are carbonaceous structures used in the field of electrochemical sensors due to their structural and physicochemical properties. Graphene and its partially reduced oxide have high electronic mobility, high electronic transfer speed, and a large surface to volume ratio, which makes them very useful in the development of electrochemical sensors [47,48]. The combination of MIPs on electrodes modified with partially reduced graphene oxide opens the opportunity to develop sensitive and selective electrochemical sensors.

In this work, we develop an electrochemical sensor based on the use of MIPs to determine paracetamol in pharmaceutical samples without pretreatment as a new control tool. The electrochemical sensor was based on a glassy carbon electrode modified with reduced graphene oxide dispersion + electrosynthetized polymer obtained from o-aminophenol. Very good analytical performance was obtained compared to a different method to detect and quantify paracetamol.

## 2. Materials and Methods

### 2.1. Reagents

PAR, H_2_SO_4_, and NaOH were obtained from Sigma–Aldrich (Darmstadt, Germany). HClO_4_, ethanol, methanol, and acetic acid were obtained from Merck p.a. Acetonitrile (ACN; HPLC grade) was obtained from Sintorgan. Ultrapure water (ρ = 18 MΩ cm) was obtained from a Millipore-Milli Q system. PAR stock solutions at concentrations of 1 and 0.1 mM were prepared daily in 0.1 M HClO_4_ solution. These solutions were kept at 4 °C and protected from light.

To detect PAR in pharmaceutical samples, different commercial drugs from diverse manufacturers were purchased. Then they were stored in a refrigerator at 4 °C before analysis.

### 2.2. Instrumentation and Electrochemical Techniques

An Epsilon potentiostat (BASi-Bioanalytical System and run with the BAS Epsilon EC Windows software version 1.60.70) coupled to a PC with incorporated analyzer software was used to perform cyclic (CV) and differential pulse (DPV) voltammetries. A C3 electrochemical cell (BASi-Bioanalytical System) with a GCE (CH Instruments, 3 mm diameter), a platinum wire, and Ag/AgCl, 3 M KCl (BAS, RE-5B) were used as working, counter, and reference electrodes, respectively, for all experiments. CV measurements were performed by applying a scan rate (υ) of 0.1 V s^−1^ in the potential window range from 0.0 V to 1.1 V. The parameters used in DPV were a step (E_step_) and amplitude (E_pulse_) of the pulse of 0.005 V and 0.050 V, respectively, and a pulse width (t_pulse_) of 40 ms, allowing υ = 0.010 V s^−1^. All DPV measurements were performed in the potential window from 0.0 V to 1.0 V.

Electrochemical impedance spectroscopy (EIS) was carried out using a PalmSens4 Potentiostat coupled to a PC with PS Trace 5.11 Software (Palm Sens, Houten, The Netherlands). To perform EIS, a sinusoidal wave with an amplitude of 0.015 V was applied in the frequency range from 50 mHz to 0.5 MHz centered at the equilibrium potential of the system (at about 0.230 V), formed by a solution of 1 mM ferrocenemethanol + 0.1 M KCl.

Scanning electron microscopy (SEM) was performed using a ΣIGMA field emission scanning electron microscope (FE-SEM) from Zeiss (Oberkochen, Germany).

PAR quantification was also performed using HPLC with a Waters 2489 Separations Module chromatograph (Manchester, United Kingdom) coupled to a binary pump (Waters 1525) and a diode array detector set at 230 nm. A PhenoSphere reverse-phase C18 column (5 μm, 250 × 4.6 mm i.d.) from Phenomenex was used to detect PAR in isocratic mode using a mobile phase consisting of ACN/H_2_O (55–45%), keeping a flow rate of 0.8 mL min^−1^ [49]. The temperature was 25.0 ± 0.1 °C.

### 2.3. Formation of the Reduced Graphene Oxide Dispersion

Graphene oxide (GO) was synthesized from graphite flakes following a previously developed methodology [50]. Subsequently, GO was partially reduced (rGO). To achieve this goal, a volume of 10 mL of graphene oxide (GO) dispersion at a concentration of 0.5 mg mL⁻¹ was placed in a glass container and combined with 0.75 mL of ammonia solution (28% *w*/*w*). Subsequently, 0.02 mL of hydrazine hydrate (50% *w*/*w*) was introduced, and the resulting mixture was stirred for 10 min. The suspension was then subjected to thermal treatment in a water bath at 60 °C for 3.5 h. After cooling to ambient temperature, the resulting material was centrifuged at 12,000 rpm for 10 min and thoroughly washed with distilled water to eliminate any residual hydrazine [51]. In order to obtain a homogeneous and stable dispersion, 3 mg of rGO was dispersed in 1 mL of 1:1 solution of 1,3-dioxolane + water in an Eppendorf tube. The mixture was manually agitated and then sonicated for 20 min to form the dispersion.

### 2.4. Preparation of the MIP-Based Electrochemical Sensor

Prior to modification of the GCE, it was polished with alumina slurries (0.30 and 0.05 µm), sonicated in a water bath for 30 s, and dried under a nitrogen stream. Then, 5 µL of rGO dispersion was added onto the GCE and dried for 30 min at 37 °C, forming the rGO/GCE. Next, the rGO/GCE was immersed in a solution of 2 mM o-AP + 10 mM PAR in an aqueous solution of 0.1 M HCIO_4_, and successive cycles of cyclic voltammetry were performed between 0 and 1.1 V at υ = 0.100 V s^−1^, where co-polymerization was produced on the rGO/GCE, forming the PAR-MIP/rGO/GCE. Next, specific cavities for PAR were formed through incubation of the PAR-MIP/rGO/GCE in an H_2_O bath for 3 h, followed by 10 cycles of CV in 0.5 M H_2_SO_4_ from 0 to 1.1 V at a υ = 0.100 V s^−1^. In addition, the PAR-MIP/rGO/GCE was immersed again in an H_2_O bath for 30 min and, finally, 5 cycles of CV under the same conditions described above. In this way, PAR was removed from co-polymer.

PAR was determined after immersion of the MIP/rGO/GCE in solutions of PAR in the concentration range of 30 to 200 nM. Confirmation of the developed MIP sensor was carried out using non-imprinted polymer (NIP), which was prepared following the same procedure as the MIP, but without adding PAR to the solution in the polymerization step.

### 2.5. Sample Preparation

Four different pharmaceutical formulations (denominated PF1, PF2, PF3, and PF4) were finely milled, transferred to a volumetric flask, and dissolved in 10 mL of 0.1 M HCIO_4_ solution. These solutions were sonicated for 60 s and then filtered. The sample solutions were used for the detection of PAR using the MIP/rGO/GCE and HPLC.

## 3. Results and Discussion

**Scheme 1:**
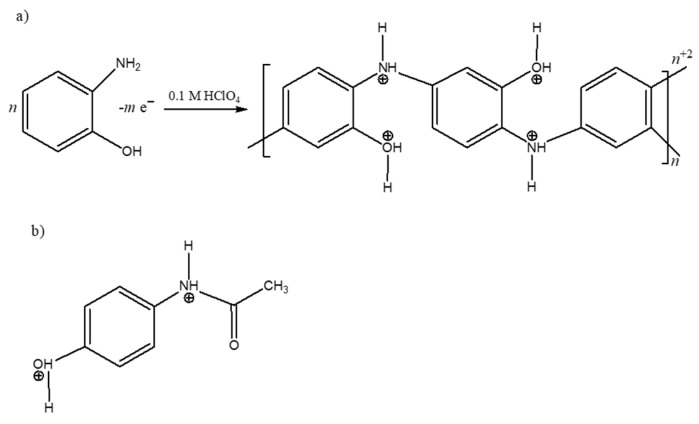
Molecular structures of (**a**) polymer obtained by electropolymerization of o-AP, and (**b**) PAR, both in 0.1 M of HClO_4_.

o-AP was selected as the monomer for polymerization due to its structural affinity with PAR. Both molecules contain nitrogen and oxygen atoms in their chemical structures, as well as an aromatic ring, suggesting the presence of two primary types of interactions, hydrogen bonding and London dispersion forces, in addition to dipole-dipole interactions. Since the polymerization was performed in a strongly acidic medium, both the synthesized polymer and PAR became partially or fully protonated (Scheme 1), leading to electrostatic repulsion. Consequently, the formation of the PAR-MIP complex could be attributed to a balance of attraction and repulsion interactions, which are highly dependent on both the solution pH and the ionic strength of the medium. Therefore, the MIP synthesis, characterization, and PAR desorption steps were investigated as described below.

### 3.1. Surface Characterization of MIP/rGO/GCE Sensor

**Figure 1:**
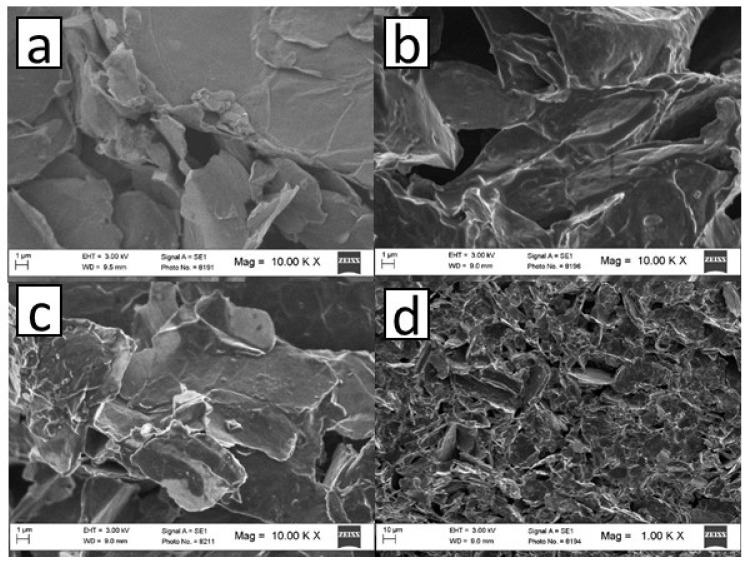
SEM images obtained at 10000X for (**a**) rGO/GCE, (**b**) MIP/rGO/GCE, and (**c**) NIP/rGO/GCE. (**d**) SEM image obtained at 1000X for MIP/rGO/GCE.

SEM of the modified electrodes was performed to analyze the modification steps described in Section 2.4. As can be observed in Figure 1a, when the GCE was modified with rGO dispersion, large overlapping rGO sheets were uniformly spread over the electrode, which formed a support for the subsequent electropolymeric generation of the MIP. When the polymer was formed in the presence (MIP/rGO/GCE) and absence (NIP/rGO/GCE) of PAR (Figure 1b,c), an increase in brightness at the edges of the rGO sheets could be observed, attributed to the conductivity of the formed polymer. In addition, the uniformity of the dispersion on the electrode was retained. However, due to the low size of PAR (molecular weight of 151.16 g mol^−1^), no significant changes were observed when the polymer was formed in the presence or absence of PAR. The uniformity of the MIP/rGO/GCE can be observed in Figure 1d, where a homogeneous distribution MIP/rGO on the electrode was observed.

### 3.2. Electrochemical Characterization of MIP/rGO/GCE Sensor

**Figure 2:**
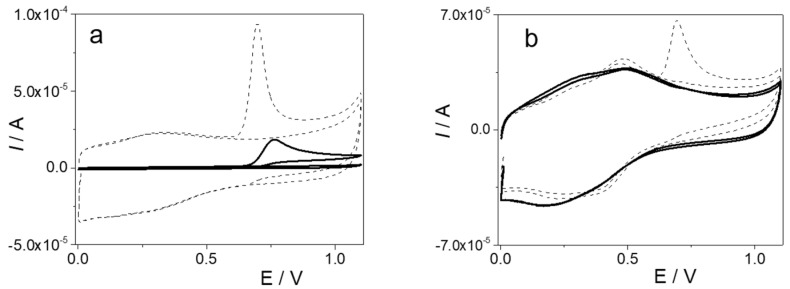
(**a**) Cyclic voltammograms obtained in 0.5 mM PAR + 0.1 M HClO_4_ solution at the bare GCE (solid line) and the rGO/GCE (dotted line). (**b**) Cyclic voltammograms for the MIP/rGO/GCE (dotted line) and NIP/rGO/GCE (solid line) in 0.1 M HClO_4_ after the electrodes were incubated for 20 min in 0.1 M HClO_4_ + 0.5 mM PAR. υ = 0.05 V s^−1^.

The behavior of the electrochemical oxidation of PAR in 0.1 M HClO_4_ was studied by cyclic voltammetry for the GCE and modified electrode. Figure 2a shows the cyclic voltammograms for 0.5 mM PAR with the GCE (solid line) and the rGO/GCE (dotted line), where, in both cases, anodic waves centered around 762 mV (*I*_a,p_ = 19 uA) and 698 mV (*I*_a,p_ = 95 uA) were observed for the GCE and rGO/GCE, respectively. For both electrodes, the anodic peak showed an irreversible behavior determined by the absence of the reduction peak when the scan was reversed, in good accordance with the electrochemical behavior of phenols. In addition, an increase in capacitive current was observed due to the increase in electrochemical area when the GCE was modified with rGO. Figure 2b shows the cyclic voltammograms of PAR oxidation at the PAR-MIP/rGO/GCE (dotted line) and NIP/rGO/GCE (solid line). For both electrodes, the cyclic voltammograms were recorded in acidic solution, after which the electrodes were incubated for 20 min in a solution of 0.5 mM PAR + 0.1 M HClO_4_. For the PAR-MIP/rGO/GCE, an anodic peak around 690 mV (*I*_a,p_ = 67 uA) due to PAR oxidation was observed. On the other hand, as was expected, in the case of the NIP/rGO/GCE, no electrochemical discharge was observed. These results showed that PAR can only oxidize through the specific pores generated during electropolymerization.

Electrochemical characterization of the bare GCE and every modification process was performed by comparing the CV and EIS results in 1 mM ferrocenemethanol + 0.1 M KCl solution.

**Figure 3:**
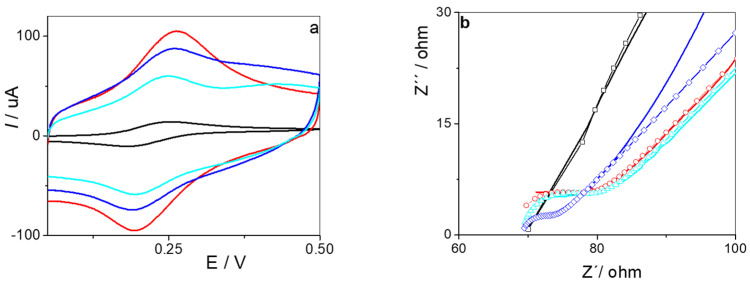
(**a**) Cyclic voltammograms obtained in 1 mM ferrocenemethanol + 0.1 M KCl solution at the GCE (black line), rGO/GCE (red line), MIP/rGO/GCE (blue light line), and NIP/rGO/GCE (blue line). (**b**) EIS plots for the GCE (black line), rGO/GCE (red line), MIP/rGO/GCE (blue light line), and NIP/rGO/GCE (blue line).

Figure 3 shows the cyclic voltammograms obtained for the different electrodes constructed. The peak current value was the lowest if there was no modification on the electrode. When the electrode was coated with a dispersion of rGO, an increase in electron transfer occurred, and a significant increase in the peak current value was observed. Once electropolymerization was performed, the conductivity of the electrodes remained higher with respect to the GCE. By comparison, the NIP/rGO/GCE exhibited a higher current response than the electrodes fabricated through polymerization of o-AP and PAR (MIP/rGO/GCE). This difference arose because the MIP contained specifically tailored cavities for PAR recognition, whereas the NIP lacked these selective binding sites.

The EIS spectra, represented by Nyquist plots, were fitted using a Randles equivalent circuit [52]. From the fitted data, we determine the charge transfer resistance (R_ct_) of ferrocenemethanol on the electrode and the double layer capacitance, which allowed us to infer the modification with rGO and the polymer. For the GCE, an R_ct_ of 72.54 Ω and a capacitance of 125.1 nF were obtained. After modification of this surface with rGO, a marked decrease in R_ct_ to 9.6 Ω was observed, accompanied by an increase in capacitance to 985.1 nF. These effects were attributed to the deposition of carbonaceous material on the surface of the GCE, which facilitated electronic transfer and increased the electroactive area, allowing a higher surface charge. When the impedance spectrum was performed using the NIP/rGO/GCE, an R_ct_ of 3.84 Ω was obtained, with this value being consistent with the deposition of a highly conductive polymer that enhances electronic mobility. On the other hand, for the MIP/rGO/GC, which presented specific cavities generated by PAR extraction, the R_ct_ increased slightly to 5.55 Ω, suggesting that the cavities affected the conductivity of the polymer. With respect to the capacitance, both the NIP and MIP/rGO/GCE showed lower values than the rGO/GCE, suggesting growth of the polymer on the rGO sheets. The results obtained by impedance spectroscopy were consistent with those observed by cyclic voltammetry.

### 3.3. Optimization of Significant Parameters in the Construction of MIP/rGO/GCE

#### 3.3.1. Determination of the Monomer-to-Template Ratio

**Figure 4:**
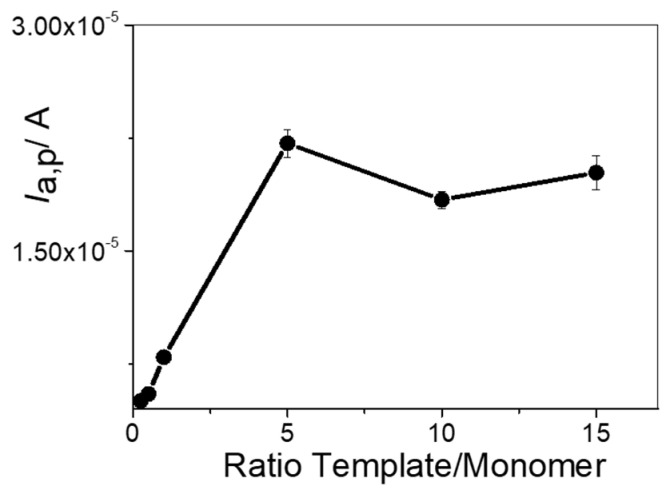
*I*_a,p_ values in 0.5 mM PAR + 0.1 M HClO_4_ obtained via DPV at different template to monomer ratios.

To create effective binding cavities, it is crucial to optimize the template-to-monomer (PAR to o-AP) ratio while ensuring that the polymer forms stably and uniformly. Finding the right balance allows for stronger interactions between the functional monomer and the template. If a lower concentration of monomer is used, the functional groups will not properly integrate into the final polymeric structure, leading to lower porosity and fewer selective cavities. On the other hand, using a higher concentration of monomer can form random distributions within the polymer rather than forming well-defined cavities. To evaluate this effect, we analyzed the *I*_a,p_ values for PAR oxidation, which represented the difference in peak currents before and after template removal. Different template-to-monomer ratios (0.25:1, 0.5:1, 1:1, 5:1, 10:1, and 15:1) were assayed at a fixed PAR concentration of 10 mM. Once the PAR-MIP/rGO/GCE was formed, PAR was removed, as described in Section 2.4, and then PAR was incubated through immersion of the MIP/rGO/GCE in 0.5 mM PAR + 0.1 M HClO_4_ solution. As can be seen from Figure 4, the *I*_a,p_ value increased as the template-to-monomer ratio increased. This phenomenon occurred up to a ratio of 5:1, after which the *I*_a,p_ signal did not show significant changes. Based on the results, the optimal ratio of functional template to monomer (PAR to o-AP) was 5:1. This ratio was then used in the following experimental steps.

#### 3.3.2. Effect of Number of Cycles of Polymerization

**Figure 5:**
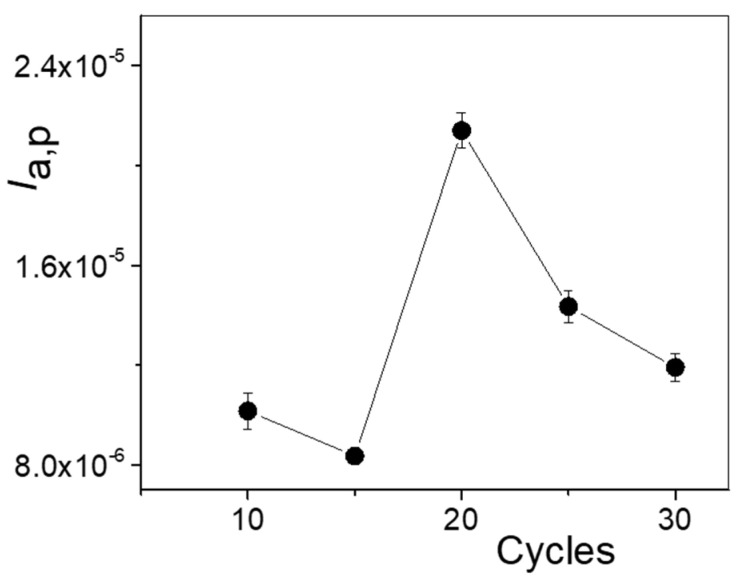
*I*_a,p_ values in 0.5 mM PAR + 0.1 M HClO_4_ obtained via DPV as a function of the number of polymerization cycles.

The cycling step is critical to form the polymer film in the presence of PAR. Numbers of cycles of cyclic voltammetry in the potential window of 0.0 V to 1.1 V from 10 to 30 were performed to fabricate the PAR-MIP/rGO/GCE. Once the template was removed, PAR was incubated on the MIP/rGO/GCE in a solution of 0.5 mM PAR + 0.1 M HClO_4_. Figure 5 shows the variation in *I*_a,p_ with the number of cycles of polymerization for MIP formation.

At a number of cycles lower than 20, far fewer specific cavities for PAR were obtained compared to a higher number of cycles. This small number of cavities implied a lower *I*_a,p_ value, and, on the other hand, the polymer formed had lower reproducibility. When the number of cycles increased from 10 to 20, an increase in *I*_a,p_ values was observed; however, for higher numbers of cycles, the current decreased sharply. Twenty cycles formed a thickness that was adequate to generate the cavities and release PAR during the removal step, as well as their specific interaction in the incubation step. Thus, 20 cycles of cyclic voltammetry was chosen for the next experiments.

#### 3.3.3. Optimization of Removal Conditions of the Template

**Figure 6:**
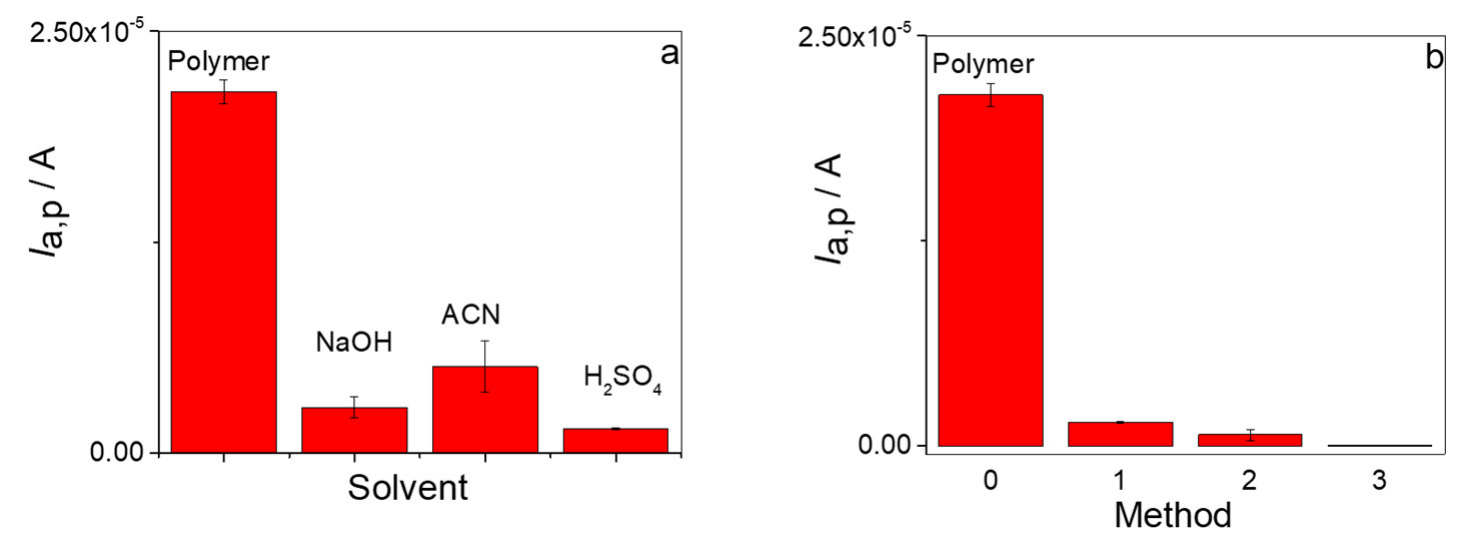
(**a**) Effectiveness of PAR removal from the polymeric matrix with respect to the type of solvent. (**b**) Effectiveness of PAR removal from the polymeric matrix with respect to the method used using H_2_SO_4_ as the main solvent.

Another factor of great significance in the formation of the MIP/rGO/GCE is the removal of the analyte from the cavities formed in the electropolymerization stage. This process aims to extract unreacted monomer residues and release PAR from the cavities formed in the polymer without affecting their structure and functionality. As interactions between PAR and the MIP are dependent on pH and ionic strength, different media were studied to optimize the desorption of PAR. In the first step, the template removal procedure was performed for 30 min using three removal solutions, such as 1 M NaOH aqueous solution, ACN, and 0.5 M H_2_SO_4_ aqueous solution. The effectiveness of the different methods used to remove PAR was evaluated in terms of *I*_a,p_ (in the potential zone around 664 mV) after the removal step, compared to *I*_a,p_ obtained for the polymer generated with 0.5 mM PAR. The lower *I*_a,p_ value obtained after each method used allowed us to infer the more effective process for cleaning and removing PAR from the polymer. Figure 6a shows a diagram bar where the *I*_a,p_ values for PAR oxidation were compared for the three solutions. When the removal step was performed with 0.5 M H_2_SO_4_ aqueous solution, the *I*_a,p_ value decreased 93.5%, showing the best performance for removing PAR. These results were consistent with both the increased proton concentration and higher ionic strength of the removal solution. In order to improve this process, three different methods for the removal of the analyte from the polymer cavities using 0.5 M H_2_SO_4_ as the principal solvent were carried out. The first method consisted of immersing the electrode in 0.5 M H_2_SO_4_ for 30 min (method 1), and then performing cyclic voltammetry in 0.5 M H_2_SO_4_, as previously described. The second method consisted of immersing the electrode in H_2_O for 12 h and then performing 10 cycles in 0.5 M H_2_SO_4_ (method 2). The third method consisted of immersion of the electrode in H_2_O for 3 h, followed by 10 cycles in 0.5 M H_2_SO_4_, then immersion of the electrode in H_2_O for 30 min and, finally, 5 cycles in 0.5 M H_2_SO_4_ (method 3).

As can be seen from Figure 6b, the addition of steps containing H_2_O as a second removal solvent and H_2_SO_4_ cycling steps greatly improved the PAR removal uptake from the polymer. Thus, 97% removal for method 2 and 99.9% removal for method 3 were obtained, which was a marked improvement over that obtained for method 1 (93.5% removal). In conclusion, PAR removal from the MIP was facilitated by two key factors: first, the electrochemical conditioning effect of cyclic voltammetry in acidic media, and second, the abrupt ionic strength change that occurred when the electrode was transferred to aqueous solution. Therefore, method 3 was chosen as the procedure to perform removal of the template from the polymer and generate the MIP again.

#### 3.3.4. Determination of the Incubation Time

**Figure 7:**
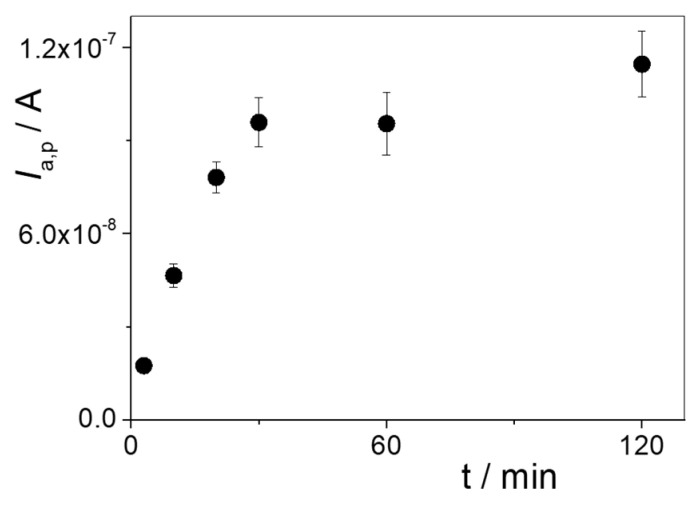
*I*_a,p_ values obtained via DPV as a function of rebinding time in 30 nM PAR + 0.1 M HClO_4_.

The incubation time to produce specific binding between PAR and the MIP was optimized. Different incubation times were assayed in 30 nM PAR + 0.1 M HClO_4_ solution under the above conditions. Figure 7 shows that the peak current for PAR oxidation continuously increased as the incubation time of PAR on the MIP/rGO/GCE increased. This growth stabilized after 30 min of incubation time; therefore, 30 min was used for PAR determination.

### 3.4. Application of MIP/rGO/GCE for the Determination of PAR

**Figure 8:**
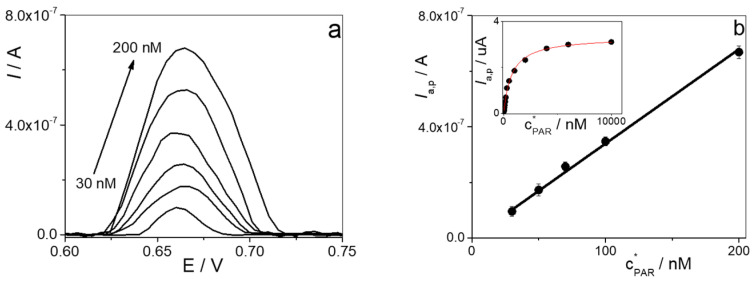
(**a**) Differential pulse voltammograms obtained at different PAR concentrations. (**b**) Calibration curve obtained for PAR in 0.1 M HClO_4_ solution. Insert: saturation curve *I*_a,p_ vs. $c_{PAR}^{*}$.

Once all parameters were optimized for PAR determination with the electrochemical sensor, a calibration curve was constructed using DPV for different concentrations of PAR in 0.1 M HClO_4_. Figure 8a shows the differential pulse voltammograms obtained for PAR concentrations lower than 200 nM. The calibration curve shown in Figure 8b exhibited a linear relationship between *I*_a,p_ and PAR concentration in the range from 30 to 200 nM, with a correlation coefficient (r^2^) of 0.9968. The analytical sensitivity was (3.4 ± 0.1) A M⁻¹, and the limit of detection (LOD), calculated as 3σ/S (where σ is the standard deviation of the blank and S is the sensitivity of the calibration curve), was 10 nM [53].

When the PAR concentration increased significantly, the current remained approximately constant (see insert in Figure 8b). The MIP-PAR binding affinity was further evaluated based on this saturation curve. Equilibrium-based analysis offers a straightforward and rapid approach for extracting affinity constants from experimental data. In this context, assuming that the *I*_a,p_ value was directly proportional to the quantity of the MIP-PAR complex formed, the interaction was modeled using the Langmuir adsorption isotherm (Equation (1)) to describe the corresponding equilibrium process [54]:

(1) $$I_{a,p}=\;I_{a,p}^{max}\frac{K_{af}c_{PAR}^{*}}{1+\;K_{af}c_{PAR}^{*}}$$

where $I_{a,p}$ is the current value obtained for each PAR concentration, $I_{a,p}^{max}$ is the current value obtained in the region at high PAR concentration, and $K_{af}$ is the affinity constant of the MIP–PAR complex. Using this equation, a $K_{af}$ = (1.35 ± 0.08) × 10^6^ M^−1^ value was obtained. The $K_{af}$ calculated was in good agreement with the literature value reported for another MIP–PAR system, which was 8.31 × 10^6^ M^−1^ [55].

### 3.5. Repeatability, Reproducibility, Stability, and Reusability Tests

Experiments were carried out using the MIP/rGO/GCE sensor to determine its stability over time. The response of the sensor in the determination of PAR at a concentration of 100 nM PAR was determined on different days using the same electrochemical sensor. Before its use, PAR was removed as previously described and stored dry at 4 °C. It was observed that PAR detection was stable for 15 days (with a decrease in the current response of less than 4%), after which a progressive reduction in the current response until to 20% was observed. Thus, the MIP/rGO/GCE sensor could be used for 15 days. The evaluation of the reusability of the MIP/rGO/GCE consisted of its use in the determination of 100 nM PAR and subsequent cleaning, as described above. In this way, each MIP/rGO/GCE was used at least 20 times. The results demonstrated that the developed sensor exhibited reusability, distinguishing it from disposable detection systems. This represents a significant advantage over conventional affinity biosensors in terms of cost effectiveness and operational sustainability.

The reproducibility of the MIP/rGO/GCE sensor was evaluated by comparing the sensitivity values obtained from calibration curves constructed using five independently fabricated electrodes. The resulting percentage variation coefficient (RSD) was 4%, indicating good inter-sensor reproducibility. Repeatability was assessed by constructing five calibration curves using the same modified electrode, yielding an RSD of 3%, which demonstrated the high stability and precision of the sensor response under repeated measurements.

**Figure 9:**
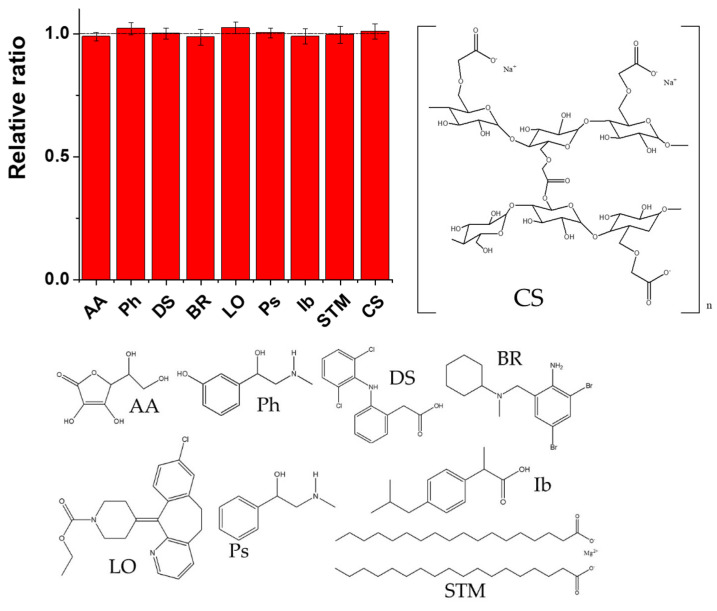
Selectivity study in the determination of PAR in the presence of potential interferents AA, Ph, DS, BR, LO, Ps, Ib, STM, and CS, presented together with their chemical structures.

Additionally, the selectivity of the sensor for the quantitation of PAR was determined in the presence of possible interfering compounds, such as AA, phenylephrine (Ph), DS, bromhexine (BR), loratadine (LO), pseudoephedrine (Ps), Ib, stearate magnesium (STM), and croscarmellose sodium (CS), at 10-fold concentrations as compared to that of PAR (at a PAR concentration of 100 nM). As shown in Figure 9 the presence of these interfering compounds caused negligible changes in the response (less than 3%).

### 3.6. Quantification of PAR in Pharmaceutical Samples

The MIP/rGO/GCE sensor was applied to quantify PAR in four pharmaceutical formulations, as outlined in Table 1. For comparison, the PAR levels in these samples were also analyzed by HPLC, as described in Section 2.2. The calibration curve for the HPLC method was constructed by plotting peak area vs. PAR concentration, yielding the following parameters: intercept = (6.5 ± 0.5) A.U., slope = (6.3 ± 0.5) A.U./M, and correlation coefficient r = 0.9942.

Table 1 presents the concentrations of PAR obtained for both the HPLC method and the electrochemical sensor for four pharmaceutical products. The values obtained from both techniques were close to the values provided by the manufacturers. These results allowed us to infer that the electrochemical sensor can be used to determine PAR in pharmaceutical samples.

**Figure 10:**
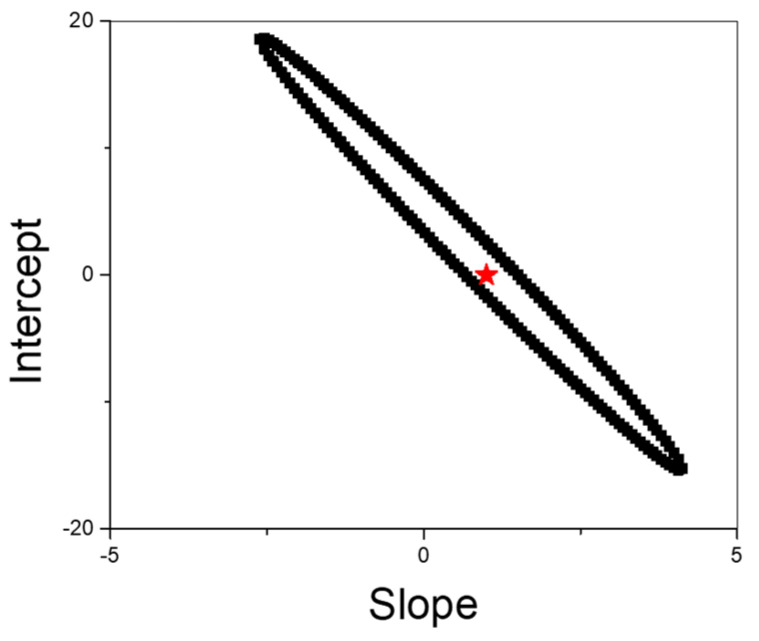
EJRC for comparison of results obtained between HPLC and the MIP/rGO/GCE sensor.

Additionally, a statistical comparison between the MIP/rGO/GCE sensor and HPLC data was carried out using the Elliptical Joint Confidence Region (EJRC) approach [56]. EJRC is particularly useful for detecting significant discrepancies across varying concentration levels [56]. As depicted in Figure 10, the EJRC encompassed the point (1,0), suggesting no statistically significant difference between the two methods at a 95% confidence level. These findings supported the conclusion that the electrochemical sensor is a reliable tool to determine PAR in pharmaceutical samples in an expensive and simple way.

The electrochemical sensor exhibited satisfactory analytical performance and was subsequently applied to the determination of PAR in pharmaceutical formulations. The main performance metrics obtained with the MIP/rGO/GCE sensor were compared with those reported in the literature for other electrochemical sensors based on MIPs as the PAR-recognition element, employing DPV as the detection technique (see Table 2).

The MIP/rGO/GCE sensor exhibited a highly competitive limit of detection (LOD) compared to the sensors reported in Table 2. It demonstrated clear potential for at least 20 reuses per sensor. Its fabrication process was robust and straightforward, avoiding complex synthesis steps or expensive hybrid platforms. The sensor provided a linear response at low concentrations, making it ideal for trace detection and applications requiring high sensitivity.

It also showed promising temporal stability, maintaining performance for approximately 15 days after initial use. Based on these features, the MIP/rGO/GCE sensor stands out for its high sensitivity and reusability, making it well suited for the accurate detection of low paracetamol concentrations in controlled pharmaceutical matrices. With an acceptable working range and operational stability, its balance of simplicity, selectivity, and cost effectiveness makes it highly competitive when compared to more complex and expensive sensor systems.

## 4. Conclusions

A glassy carbon electrode modified with an electropolymerized o-aminophenol film enabled the spontaneous accumulation and sensitive detection of paracetamol. The developed electrochemical sensor demonstrated excellent analytical performance, including a low detection limit (10 nM), good reproducibility (4%) and repeatability (3%), and successful quantification of four pharmaceutical formulations, with the results in excellent agreement with HPLC data. Its simplicity, low fabrication cost, and reusability (up to 20 measurements per day with at least 15 days of stability) make it a promising alternative to chromatographic techniques, especially for portable and on-site applications. Furthermore, its applicability to complex real matrices suggests the potential extension of this platform to the analysis of other analytes of interest.

## Figures and Tables

**Table 1:** Concentrations of PAR determined in four pharmaceutical formulations using the electrochemical sensor and HPLC.

Sample	Reported by the Manufacturer mg PAR/Sample	Detection Method		Recovery %
		HPLC	MIP/rGO/GCE	MIP/rGO/GCE
M1	1000	1015	1000	100
M2	500	572	466	93
M3	650	640	656	101
M4	503	526	502	100

**Table 2:** Comparison of analytical performance parameters of the MIP/rGO/GCE sensor with previously reported electrochemical sensors for PAR determination.

Platform	Linear Range (µmol/L)	LOD (µmol/L)	Reusability	Stability (Days)	Reference
MIP(PCz-co-PPy)/ AuPd/GN-CNTs-IL	0.10−10	0.05	n.r. *	30	[27]
NanoMIPs/SPCE	100−1000	70	n.r.	180	[28]
ZrO_2_-MIP-Au/rGO	0.01−10; 10−400	0.01	13 uses	40	[29]
MIP/MoS_2_/CNTs/GCE	0.01−300	0.003	n.r.	15	[30]
MMIP/MCPE	0.06−50; 50−200	0.0173	n.r.	30	[31]
MIP/GO@COF/GCE	0.05−20	0.032	n.r.	30	[32]
MIP/rGO/GCE	0.03−0.2	0.010	20 uses	15	This work

* n.r. = not reported.

## Data Availability

The original contributions presented in this study are included in the article. Further inquiries can be directed to the corresponding authors.
